# Sensorimotor Behavior in Individuals with Autism Spectrum Disorder and Their Unaffected Biological Parents

**DOI:** 10.21203/rs.3.rs-2973214/v1

**Published:** 2023-05-25

**Authors:** Erin K. Bojanek, Shannon E. Kelly, Lauren M. Schmitt, Stormi P. White, John A. Sweeney, Andreas Sprenger, Kathryn E. Unruh, Matthew W. Mosconi

**Affiliations:** University of Rochester, University of Rochester Medical Center; Scholars Strategy Network; Cincinnati Children’s Hospital Medical Center; Emory University School of Medicine; University of Cincinnati College of Medicine; University of Lübeck; University of Kansas; University of Kansas

**Keywords:** autism spectrum disorder, sensorimotor, broader autism phenotype, familiality, endophenotype, heritable risk

## Abstract

**Background:**

Sensorimotor impairments are common in autism spectrum disorder (ASD) and evident in unaffected first-degree relatives, suggesting that they may serve as important endophenotypes associated with inherited risk. We tested the familiality of sensorimotor impairments in ASD across multiple motor behaviors and effector systems and in relation to parental broader autism phenotypic (BAP) characteristics.

**Methods:**

Fifty-eight autistic individuals (probands), 109 parents, and 89 control participants completed tests of manual motor and oculomotor control. Sensorimotor tests varied in their involvement of *rapid*, feedforward control and *sustained*, sensory feedback control processes. Subgroup analyses compared families with at least one parent showing BAP traits (BAP+) and those in which neither parent showed BAP traits (BAP-).

**Results:**

Probands with BAP- parents (BAP- probands) showed rapid manual motor and oculomotor deficits, while BAP+ probands showed sustained motor impairments compared to controls. BAP- parents showed impaired rapid oculomotor and sustained manual motor abilities relative to BAP+ parents and controls. Atypical rapid oculomotor impairments also were familial.

**Limitations:**

Larger samples of ASD families including greater samples of probands with BAP+ parents are needed. Genetic studies also are needed to link sensorimotor endophenotype findings directly to genes.

**Conclusions:**

Results indicate rapid sensorimotor behaviors are selectively impacted in BAP- probands and their parents and may reflect familial liabilities for ASD that are independent of familial autistic traits. Sustained sensorimotor behaviors were affected in BAP+ probands and BAP- parents re ecting familial traits that may only confer risk when combined with parental autistic trait liabilities. These findings provide new evidence that rapid and sustained sensorimotor alterations represent strong but separate familial pathways of ASD risk that demonstrate unique interactions with mechanisms related to parental autistic traits.

## Introduction

Autism spectrum disorder (ASD) is etiologically diverse. Highly penetrant but rare gene variants have been implicated in up to 20% of cases [1, 2], and several environmental factors also confer risk [3]. Still, ASD is highly heritable, and a plurality of cases involve multiple common allelic variants that individually contribute only incremental risk [4–6]. These common variants are difficult to detect given their small individual effects and variability across affected individuals. Understanding of pathogenic processes associated with majority of cases thus remains limited but could be advanced by identifying downstream traits more strongly associated with causal mechanisms than diagnostic status.

Establishing endophenotypes, or quantitative trait dimensions that are not directly observable, associated with affectation status, and related to genetic risk for a condition, can offer a powerful method for clarifying pathogenicity [7, 8]. Few endophenotypes have been identified in ASD, though several trait dimensions have shown promise for assessing familial risk. For example, “broader autism phenotypic” (BAP) traits, including social aloofness, communication difficulties, and rigid personality traits, are more prevalent in unaffected family members of autistic individuals relative to the non-autistic population [9–12]. Cognitive processing deficits, including reduced behavioral flexibility and inhibitory control, also are associated with core autism traits and disrupted in unaffected parents compared to age-matched controls [13]. Importantly, behavioral inflexibility is more severe in parents *with* BAP features and their autistic children relative to parents *without* BAP features, suggesting that these neurocognitive phenotypes and BAP traits overlap in families to confer risk for ASD [13]. The relationships between separate candidate endophenotypes associated with ASD seldom have been studied but may be critical to clarifying mechanisms of heritable risk for ASD.

Sensorimotor differences represent promising endophenotypes for ASD as they are highly quantifiable, highly heritable [14, 15], and evident in autistic individuals [16, 17] and their unaffected relatives [18, 19]. Our group previously demonstrated, in a separate sample, that rapid sensorimotor behaviors supported by feedforward planning and sustained behaviors guided by sensory feedback each are impaired in autism across manual motor and oculomotor systems [17, 20–22]. We also documented similar oculomotor differences in unaffected first-degree relatives of autistic individuals [19]. These findings suggest that rapid and sustained sensorimotor behaviors may represent endophenotypes associated with familial risk for ASD. In the present study, we aimed to replicate and extend these findings in three key ways. First, we examined sensorimotor behaviors in a large sample of autistic families across manual motor and oculomotor systems. Second, to determine the extent to which familial sensorimotor and BAP traits interact to confer risk for ASD, we compared sensorimotor behaviors in families with and without parental BAP traits. Third, we tested rapid and sustained sensorimotor behaviors in family trios allowing us to assess inter-relationships across autistic individuals and their biological parents.

## Methods and Materials

### Participants

Fifty-eight autistic participants (probands; aged 5–22 years), 109 of their unaffected biological parents (proband parents; aged 29–54 years), and 89 control participants were examined (Table 1). This sample included 43 family trios (proband, biological mother and father), 15 family dyads (i.e., proband and one parent), three probands whose parents did not complete testing, and three proband parents whose child did not complete testing. Forty-two typically developing (TD) controls were matched on age, handedness, and nonverbal IQ to the probands, and 47 neurotypical adults were matched with proband parents on age, sex, handedness, and nonverbal IQ (i.e., parent controls). Due to scheduling, technical, or compliance issues, multiple individuals did not complete all sensorimotor tests (see Tables S1 and S2 for details). IQ was estimated using the Wechsler Preschool and Primary Scale of Intelligence, Fourth Edition (4 probands, 8 TD controls; 23), Differential Ability Scale, Second Edition (3 probands; 24), or Wechsler Abbreviated Scale of Intelligence, Second Edition (51 probands, 34 TD controls, 108 proband parents, and 44 parent controls; 25). Three parent controls and one proband parent did not complete IQ testing due to scheduling difficulties or limited English proficiency.

ASD diagnoses were confirmed using the Autism Diagnostic Inventory-Revised (ADI-R; 26), the Autism Diagnostic Observation Schedule, Second Edition (ADOS-2; 27), and expert clinical opinion based on DSM-5 criteria [28]. Autistic participants were excluded if they had any known genetic condition associated with ASD (e.g., Fragile X Syndrome, etc.). Control participants had a score ≤ 8 on the Social Communication Questionnaire (SCQ; 29) and no known history of psychiatric or neurological disorders, first- or second-degree relatives with ASD, or first-degree relatives with a major psychiatric disorder. Two proband parents scored higher on the SCQ (≥ 15); one completed an ADOS-2 and did not meet classification criteria for ASD, and one did not complete the ADOS-2 due to scheduling difficulties. There were no prior concerns about an ASD diagnosis, so they are included in final analyses. No participants were taking any medications known to affect sensorimotor function at the time of testing (e.g., stimulants, antipsychotics, anticonvulsants, benzodiazepines; 30) or had a history of head injury, birth injury, or seizure disorder. Adult participants provided written consent. For minors, a legal guardian’s written consent and the minor’s written assent were obtained. The study was conducted according to procedures approved by the University of Illinois at Chicago and the University of Texas Southwestern Medical Center Institutional Review Boards.

### Procedures

#### Precision Grip Tasks

During precision grip testing, participants were seated in a darkened room 52 cm from a 102 cm Samsung LCD monitor (resolution: 1366 × 768; refresh rate: 120 Hz). Participants used their thumb and index finger to press against two opposing precision load cells (ELFF-B4–100N; Entran). A Colbourn (V72–25) resistive bridge strain amplified load cell analog signals. Data were sampled at 120 Hz with a 16-bit analog-to-digital converter (DI-720; DATAQ Instruments) and converted to Newtons.

**Figure 1:**
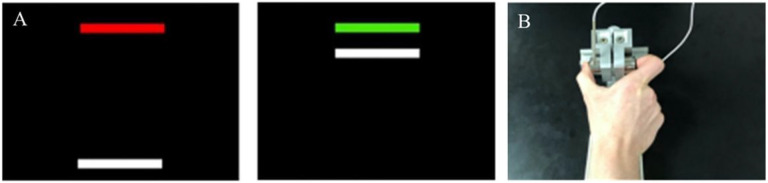
Precision gripping tasks. A. Precision gripping stimuli. Participants viewed a red target bar and white force bar. When the red target bar turned green, participants were instructed to press the load cells as quickly as possible so that the white force bar reached the level of the green target bar and continue pressing so that the white force bar maintained the level of the green target bar. B. Load cell apparatus.

Before testing, participants’ maximum voluntary contraction (MVC) was calculated for each hand using the average of their maximum force output across three, three second trials. During testing, participants viewed a white horizontal force bar that moved upwards with increased force and a static target bar (i.e., red during rest and green to cue the start of the trial; Fig.1). Participants were instructed to press the load cells when the red target bar turned green and continue pressing until the bar turned red. Participants completed “rapid” (2 seconds) and “sustained” (8 seconds) trials at 15%, 45%, and 85% of their MVC based on evidence that relative group differences vary as a function of force load [17]. The rapid test included two blocks of five trials for each hand at each force level (60 trials). The sustained test included two blocks of three trials for each hand at each force level (36 trials).

Force output for each trial was low-pass filtered via a double-pass 4th -order Butterworth filter with a cutoff of 15 Hz. Data were analyzed using custom MATLAB scripts [22]. During the rapid test, we examined the accuracy of participants’ initial force output, defined as the force at the rise phase offset divided by the target force. The rise phase offset was the timepoint when the rate of force increase first fell below 5% of the peak rate of force increase, and the force level was within 90–110% of the mean force of the sustained phase [22].

During the sustained test, we measured force output for six seconds after the rise phase offset. Force variability was examined using coefficient of variation (CoV) calculated as the standard deviation of the force time series divided by participants’ mean force. Trials in which participants stopped pressing for > 1 second or completed < 4 seconds of sustained force were excluded.

#### Oculomotor Tasks

Participants completed oculomotor testing in a darkened black room with a chinrest positioned 63 cm from a 102 cm anti-glare LCD monitor (resolution: 1920 × 1080; refresh rate: 60 Hz). Participants’ eye movements were recorded using an infrared, binocular camera-based eye tracking system with a 500 Hz sampling rate and a gaze-position error of < 0.5 degrees of visual angle (EyeLink II, SR Research Ltd., Canada).

**Figure 2:**
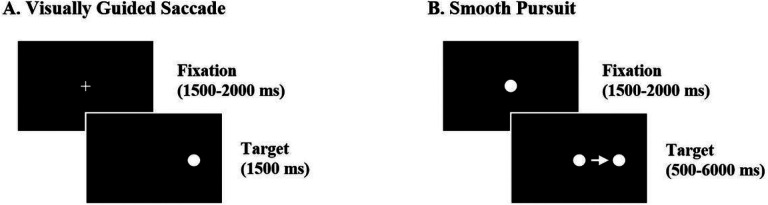
Oculomotor tasks. A. Visually guided saccade task. Participants fixated on a white central crosshair, then made saccades toward targets (i.e., white circles) which appeared at +12 or 24 degrees contemporaneously with the disappearance of the crosshair. B. Smooth pursuit task. Participants fixated on a central target (i.e., white circle), then followed the target with their gaze as the target moved from center to ±15 degrees at 2.5, 7.4, 14.9, 22.2, or 30 degrees/second.

Participants completed visually guided saccade (VGS) and smooth pursuit tasks (Fig. 2). During VGS, participants fixated on a central crosshair, then looked toward peripheral targets at ± 12 or 24 degrees of visual angle given prior findings that deficits vary across saccade amplitudes [20]. Twelve- and 24-degree targets were presented in two blocks of 30 trials. During the smooth pursuit task, participants fixated on a central target and then tracked the target from 0 to ± 15 degrees moving at 2.5, 7.4, 14.9, 22.2, or 30 degrees/second. Participants completed 40 interleaved trials (4 trials per direction per velocity). A five-point calibration was completed prior to oculomotor testing and a 3-point drift correction was completed before each subsequent block of trials.

Digital finite impulse response filters with non-linear transition bands were applied to the eye movement data prior to scoring. For the VGS test, saccade onset and offset were identified as the timepoints when eye velocity exceeded and fell below 30 degrees/second, respectively. The accuracy, peak velocity, and duration of the primary saccade (i.e., the first saccade that moved at least 20% of the distance to the target) were examined. Accuracy was the absolute value of the horizontal distance in degrees of visual angle between the final saccade location and the target location [20]. Saccades with latencies ≤ 70 ms were excluded from analyses. To control for differences in saccade amplitude, we divided peak velocity and duration by saccade amplitude. For the smooth pursuit task, pursuit onset was defined as the timepoint when eye gaze exceeded 2 degrees/second for 20 ms. Non-pursuit eye movements (e.g., saccades, blinks) and artifacts (e.g., large head movements) were removed. The ratio of pursuit velocity to the target velocity (i.e., pursuit gain) was examined.

### Clinical Measures

Symptoms related to ASD were assessed using the ADOS-2 calibrated severity scale (CSS; 31,32) and ADI-R algorithm scores. The Repetitive Behavior Scale-Revised (RBS-R; 33) was used to measure repetitive behaviors. The Conners Parent Rating Scale (Conners-3; 34) was used to measure inattention and hyperactivity/impulsivity. Subclinical ASD features were assessed in parents using the Broad Autism Phenotype-Questionnaire (BAP-Q; 101 self-report, 3 spouse report; 35). Parents were identified as “BAP+” if they exceeded the threshold for any BAP-Q subscale based on published cutoffs (9; see Table 2 for details). Probands with at least one BAP + parent were identified as “BAP+” (N = 22; including four probands with two BAP + parents, 17 probands with one BAP + and one BAP- parent, and one proband with one BAP + parent and one parent who did not complete the BAP-Q). Probands with two BAP- parents were categorized as “BAP-” (N = 23). Probands with missing BAP-Q data from at least one parent were excluded from BAP+/− subgroup analyses (N = 11) unless their parent completing the assessment met BAP + criteria. Handedness was assessed using the Physical and Neurological Examination for Soft Signs (PANESS; 36). Participants’ writing hand was identified as their dominant hand if they showed mixed handedness on the PANESS.

### Statistical Analyses

Separate linear multilevel models (MLMs) were used to examine sensorimotor behaviors in probands and parents. MLMs examining precision gripping performance included trial effects (i.e., force level, hand [dominant vs non-dominant]) as level 1 predictors and subject effects (i.e., group [proband or proband parent vs control], age, sex) as level 2 predictors. Force level was linearly transformed with 15% MVC set to zero. Based on findings that sensorimotor behaviors show a protracted course of development but limited change during adulthood [17, 37], age (converted to z-scores) was included in the MLMs for proband but not parent analyses. Sex was included in parent but not proband analyses due to the small number of female probands. All dichotomous variables (e.g., hand, group) were centered using contrast coding. Random variance components for the intercept for force level and all two- and three-way interactions were included. Grip force variability (CoV) was transformed using natural log transformations due to a non-normal distribution. We compared nested models using likelihood ratio tests to determine whether each predictor improved model fit. Variables that did not improve model fit (p > 0.05) were removed. Models with significant interaction effects included all lower-level interactions and main effects of variables included in the interaction in the final models [38].

MLMs for oculomotor variables were identical to those for precision gripping variables except level 1 predictors included target direction (left vs right), target amplitude (12 vs 24 degrees), and target velocity (for smooth pursuit gain only) as well as a random slope for target velocity in the smooth pursuit MLM. Target velocity was linearly transformed with 2.5 degrees/second set to zero. To compare performance across BAP groups, similar MLMs were conducted with group defined as a factor including BAP + and BAP- probands or BAP + and BAP- parents, with each control group as the reference group. Cohen’s d values were calculated for each significant group effect or group-related interaction.

Associations between precision gripping, oculomotor behaviors, and autistic traits were examined using Spearman correlations separately for probands and proband parents. Only correlations with |r| >0.50 and p < 0.01 are reported. We examined differences in autistic traits between BAP + and BAP- probands using t-tests. The familiality of sensorimotor behaviors within family trios and duos (i.e., proband and one proband parent) was determined using the Sequential Oligogenic Linkage Analysis Routines (SOLAR; Southwest Foundation for Biomedical Research; 39) as done previously [13].

## Results

### Rapid Sensorimotor Behaviors

#### Grip Force Accuracy

**Figure 3:**
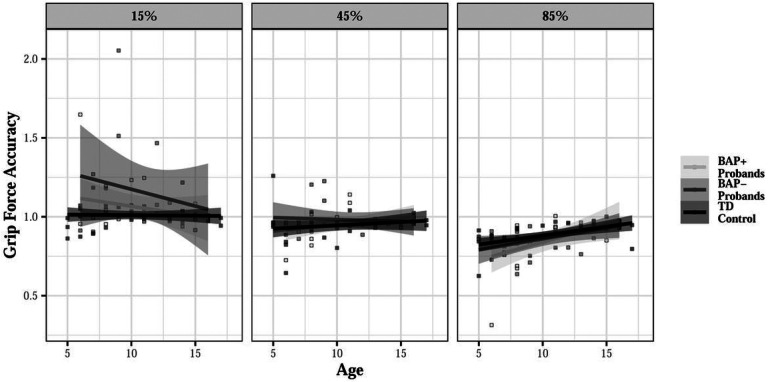
Grip force accuracy during rapid precision gripping for probands and TD controls across force levels as a function of age and broader autism phenotype (BAP) classification. During the rapid grip task, BAP-probands showed greater overshooting at 15% MVC than BAP+ probands (d=0.206) and TD controls (d=0.294), particularly at younger ages [group (BAP- vs TD Control) × age × MVC interaction: β=0.007, SE=0.002, p=0.006].

Probands showed greater force overshoot than TD controls at 15% MVC (d = 0.254), particularly at younger ages (group × age × MVC: β = 0.006, SE = 0.002, p = 0.001; Figure S1). This small but significant effect was driven by BAP- probands who showed greater force overshoot than BAP + probands (d = 0.206) and TD controls (d = 0.294) at 15% MVC at younger ages [group (BAP- vs TD control) × age × MVC: β = 0.007, SE = 0.002, p = 0.006; group (BAP + vs TD control) × age × MVC: β = 0.003, SE = 0.002, p = 0.219; BAP + vs BAP- at 15% MVC: t = 2.392, p = 0.029, Fig. 3).

Female parent controls showed greater force overshoot than proband mothers, though this was a small effect (d = 0.117). Proband fathers and male controls showed similar performance (group × sex: β=−0.020, SE = 0.008, p = 0.008; Figure S2). No differences between BAP + or BAP- parents and controls were seen for rapid force accuracy.

#### Saccade Accuracy

**Figure 4:**
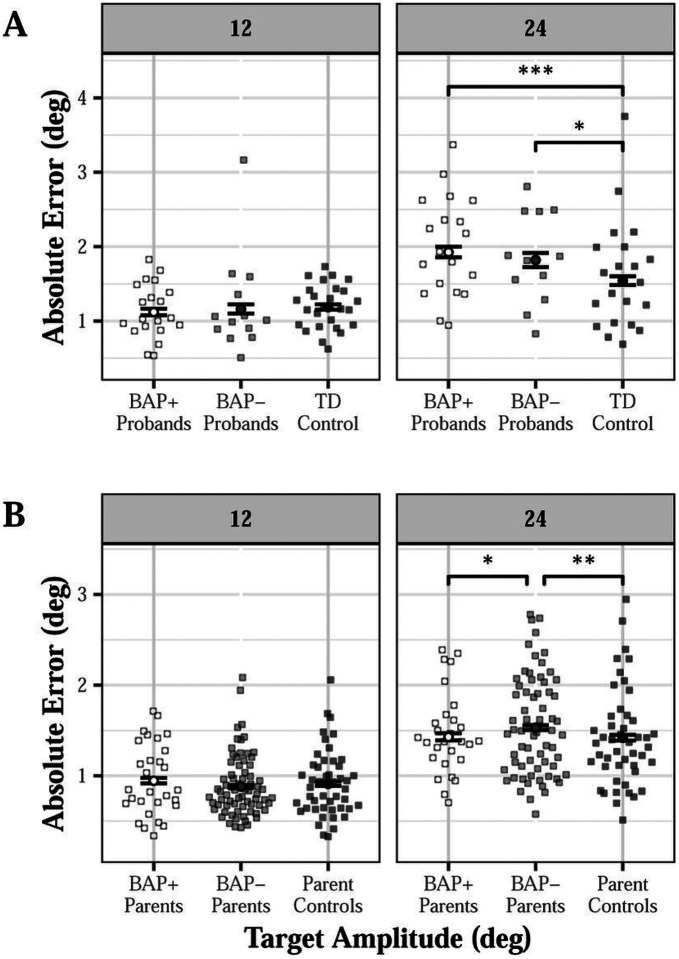
Accuracy of visually guided saccades across target step amplitudes for probands (A) and proband parents (B) as a function of broader autism phenotype (BAP) classification. BAP+ (d=0.278) and BAP-probands (d=0.196) showed increased error to 24-degree targets relative to TD controls, but not to 12-degree targets [A; group (BAP- vs TD control) × amplitude: β=0.237, SE=0.118, p=0.044; group (BAP+ vs TD control) × amplitude: β=0.341, SE=0.103, p<0.001]. BAP- parents showed increased error to 24-degree targets relative to BAP+ parents (d=0.095) and parent controls (d=0.103), but not to 12-degree targets [B; group (BAP- vs control) × amplitude: β=0.135, SE=0.046, p=0.004; group (BAP+ vs control) × amplitude: β=−0.030, SE=0.058, p=0.609]. *p<0.05. **p<0.01. ***p<0.001.

Probands showed greater saccade error relative to TD controls for 24-degree targets (d = 0.236; group × amplitude: β = 0.291, SE = 0.088, p < 0.001; Figure S3A). This difference was seen in both BAP+ (d = 0.278) and BAP- (d = 0.196) probands compared to TD controls [group (BAP- vs TD control) × amplitude: β = 0.237, SE = 0.118, p = 0.044; group (BAP + vs TD control) × amplitude: β = 0.341, SE = 0.103, p < 0.001; Fig. 4A]. For leftward saccades, BAP- probands showed greater error compared to TD controls [d = 0.103; group (BAP- vs TD control) × direction: β=−0.302, SE = 0.114, p = 0.008; group (BAP + vs TD control) × direction: β = 0.142, SE = 0.100, p = 0.154].

Proband parents showed increased saccade error relative to parent controls at 24-degrees (d = 0.084; group × amplitude: β = 0.094, SE = 0.043, p = 0.032; Figure S3B). BAP- parents showed greater error to 24-degree targets compared to BAP + parents (d = 0.095) and parent controls [d = 0.103; group (BAP- vs control) × amplitude: β = 0.135, SE = 0.046, p = 0.004; group (BAP + vs control) × amplitude: β=−0.030, SE = 0.058, p = 0.609; BAP + vs BAP- at 24-degrees: t=−2.140, p = 0.033; Fig. 4B].

#### Saccade Velocity

**Figure 5:**
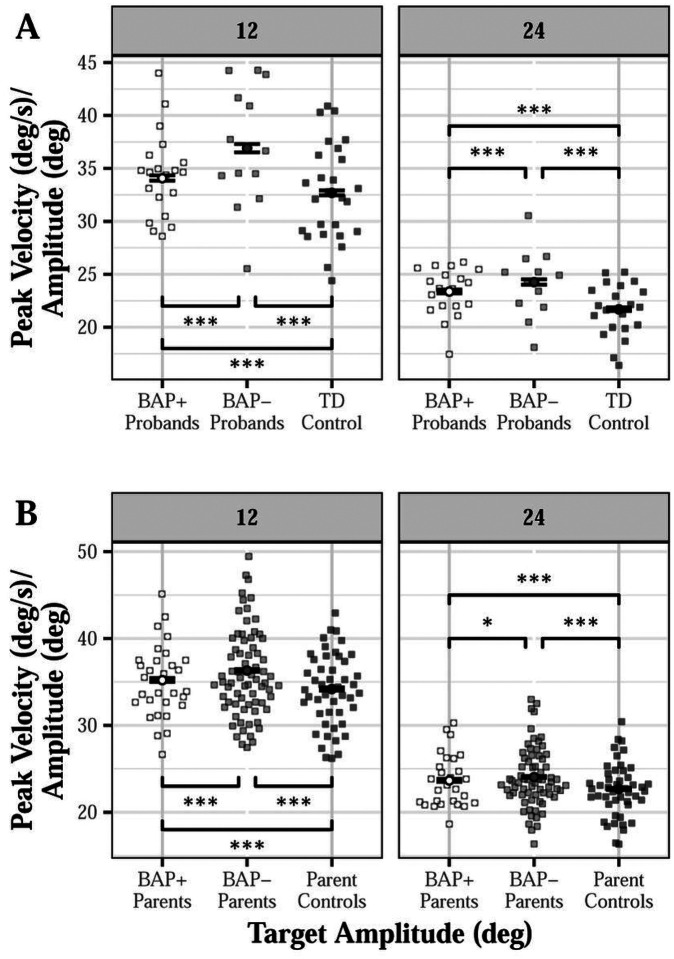
Peak velocity (after controlling for saccade amplitude) of visually guided saccades across target step amplitudes for probands (A) and proband parents (B) as a function of broader autism phenotype (BAP) classification. BAP+ probands (12°: d=0.258, 24°: d=0.486) showed higher peak saccade velocities compared to TD controls, and BAP- probands showed higher peak velocities compared to BAP+ probands (12°: d=0.475, 24°: d=0.240) and TD controls (12°: d=0.684, 24°: d=0.680), especially for 12-degree targets [A; group (BAP- vs control) × amplitude: β=−2.436, SE=0.372, p<0.001; group (BAP+ vs control) × amplitude: β=0.915, SE=0.326, p=0.005]. BAP+ parents showed higher peak saccade velocities compared to parent controls (12°: d=0.175, 24°: d=0.250), and BAP- parents showed higher peak velocities compared to BAP+ parents (12°: d=0.181, 24°: d=0.097) and parent controls (12°: d=0.352, 24°: d=0.347), especially for 12-degree targets [B; group (BAP- vs control) × amplitude: β=−0.544, SE=0.197, p=0.006; group (BAP+ vs control) × amplitude: β=−0.425, SE=0.249, p=0.088]. *p<0.05. ***p<0.001.

Probands demonstrated higher peak saccade velocities compared to TD controls, especially for leftward saccades (left: d = 0.326, right: d = 0.269; group × direction: β= −0.629, SE = 0.271, p = 0.002; Figure S4A). Both BAP+ (12°: d = 0.258, 24°: d = 0.486) and BAP- probands (12°: d = 0.684, 24°: d = 0.680) showed higher saccade velocity compared to TD controls, and BAP- probands showed higher saccade velocity than BAP + probands, especially for 12-degree targets [12°: d = 0.475, 24°: d = 0.240; group (BAP- vs TD control) × amplitude: β=−2.436, SE = 0.372, p < 0.001; group (BAP + vs TD control) × amplitude: β = 0.915, SE = 0.326, p = 0.005; BAP + vs BAP- at 12-degrees: t=−6.166, p < 0.001; BAP + vs BAP- at 24-degrees: t=−2.900, p = 0.004; Fig. 5A]. BAP + probands showed higher saccade velocity compared to TD controls only for leftward targets [d = 0.198; group (BAP- vs TD control) × direction: β=−0.097, SE = 0.358, p = 0.786; group (BAP + vs TD control) × direction: β=−0.751, SE = 0.312, p = 0.016].

Proband parents showed higher peak saccade velocity compared to parent controls, especially at 24-degrees (12°: d = 0.287, 24°: d = 0.304; group × amplitude: β= −0.501, SE = 0.185, p = 0.001; Figure S4B). BAP + parents showed increased velocity compared to parent controls (12°: d = 0.175, 24°: d = 0.250), and BAP- parents showed increased velocity compared to BAP + parents (12°: d = 0.181, 24°: d = 0.097) and parent controls (12°: d = 0.352, 24°: d = 0.347), particularly at 12-degrees [group (BAP- vs control) × amplitude: β=−0.544, SE = 0.197, p = 0.006; group (BAP + vs control) × amplitude: β=−0.425, SE = 0.249, p = 0.088; BAP + vs BAP- at 12-degrees: t=−4.258, p < 0.001; BAP + vs BAP- at 24-degrees: t=−2.067, p = 0.039; Fig. 5B].

#### Saccade Duration

**Figure 6:**
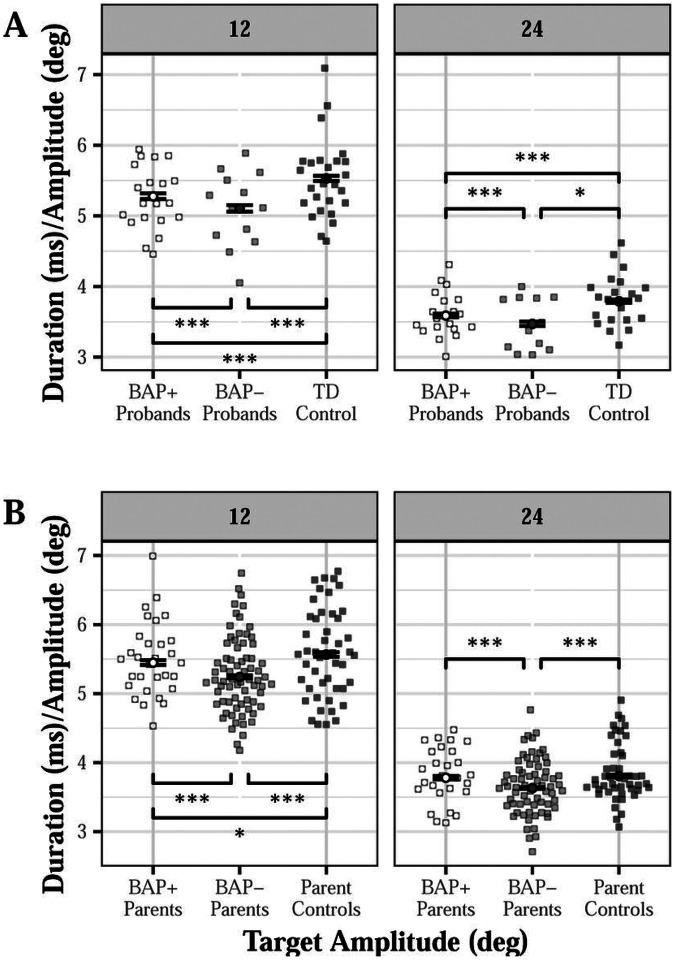
Duration (after controlling for saccade amplitude) of visually guided saccades across target step amplitudes for probands (A) and proband parents (B) as a function of broader autism phenotype (BAP) classification. BAP+ probands showed shorter saccade durations compared to TD controls (d=0.248), and BAP- probands showed shorter saccade durations compared to BAP+ probands (d=0.114) and TD controls (A; d=0.364; BAP- vs control: β=−0.419, SE=0.139, p=0.004; BAP+ vs control: β=−0.267, SE=0.120, p=0.030). BAP+ parents showed shorter saccade duration compared to parent controls for 12-degree targets (d=0.120); BAP- parents showed shorter saccade duration compared to BAP+ parents (12°: d=0.221, 24°: d=0.264) and parent controls (12°: d=0.329, 24°: d=0.326) for 12- and 24-degree targets [B; group (BAP- vs control) × amplitude: β=0.112, SE=0.0439, p=0.004; group (BAP+ vs control) × amplitude: β=0.098, SE=0.049, p=0.045]. *p<0.05. ***p<0.001.

Probands made saccades with shorter durations compared to TD controls (d = 0.266; β= −0.243, SE = 0.102, p = 0.020; Figure S5A). Both BAP+ (d = 0.248) and BAP- probands (d = 0.364) showed shorter saccade duration than TD controls, and BAP- probands showed shorter saccade duration than BAP + probands (d = 0.114; BAP- vs TD control: β=−0.419, SE = 0.139, p = 0.004; BAP + vs TD control: β=−0.267, SE = 0.120, p = 0.030; BAP + vs BAP-: t = 2.074, p = 0.038; Fig. 6A).

Proband parents showed shorter saccade durations compared to parent controls, particularly at 12-degrees (12°: d = 0.262, 24°: d = 0.213; group × amplitude: β = 0.118, SE = 0.036, p = 0.001; Figure S5B). BAP- parents demonstrated shorter saccade durations compared to BAP + parents (12°: d = 0.221, 24°: d = 0.264) and parent controls (12°: d = 0.329, 24°: d = 0.326) at 12 and 24 degrees; BAP + parents showed shorter saccade duration compared to controls only for 12-degree targets [d = 0.120; group (BAP- vs control) × amplitude: β = 0.112, SE = 0.0439, p = 0.004; group (BAP + vs control) × amplitude: β = 0.098, SE = 0.049, p = 0.045; BAP + vs BAP- at 12-degrees: t = 4.885, p < 0.001; BAP + vs BAP- at 24-degrees: t = 5.310, p < 0.001; Fig. 6B].

### Sustained Sensorimotor Behaviors

#### Grip Force Variability

**Figure 7:**
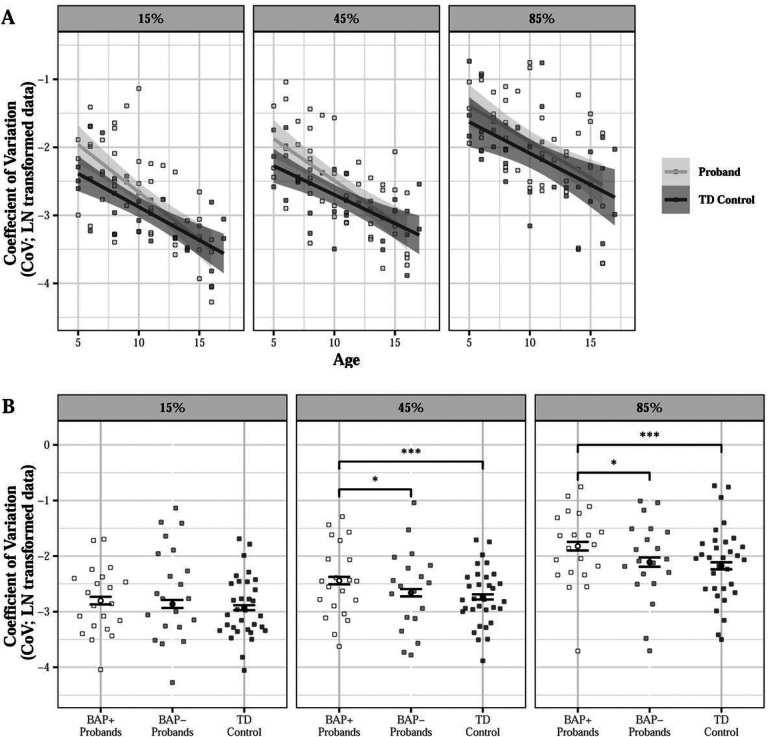
Force variability during sustained precision gripping across force levels for probands as a function of age (A) and broader autism phenotype (BAP) classification (B). During the sustained grip task, there was a significant group × age × MVC interaction. Probands showed increased sustained force variability relative to controls (15% MVC: d=0.160; 45% MVC: d=0.263; 85% MVC: d=0.222) and this was especially true at younger ages (A; group × age × MVC: β=0.011, SE=0.005, p=0.047). BAP+ probands showed increased variability compared to TD Controls (15% MVC: d=0.172; 45% MVC: d=0.386; 85% MVC: d=0.407) and BAP- probands (15% MVC: d=0.074; 45% MVC: 0.275; 85% MVC: d=0.327) at higher force levels [B; group (BAP+ vs Control) × MVC: β=0.015, SE=0.007, p=0.025]. *p<0.05, ***p<0.001.

Probands showed increased force variability relative to controls (15% MVC: d = 0.160; 45% MVC: d = 0.263; 85% MVC: d = 0.222), especially at younger ages (group × age × MVC: β = 0.011, SE = 0.005, p = 0.047; Fig. 7A). Increased force variability was driven by BAP + probands who showed increased variability relative to TD controls (15% MVC: d = 0.172; 45% MVC: d = 0.386; 85% MVC: d = 0.407) and BAP- probands (15% MVC: d = 0.074; 45% MVC: 0.275; 85% MVC: d = 0.327), especially at higher force levels [group (BAP- vs TD control) × MVC: β=−0.006, SE = 0.007, p = 0.387; group (BAP + vs TD control) × MVC: β = 0.015, SE = 0.007, p = 0.025; BAP + vs BAP- at 45% MVC: t=−2.313, p = 0.021; BAP + vs BAP- at 85% MVC: t=−2.517, p = 0.013; Fig. 7B].

**Figure 8:**
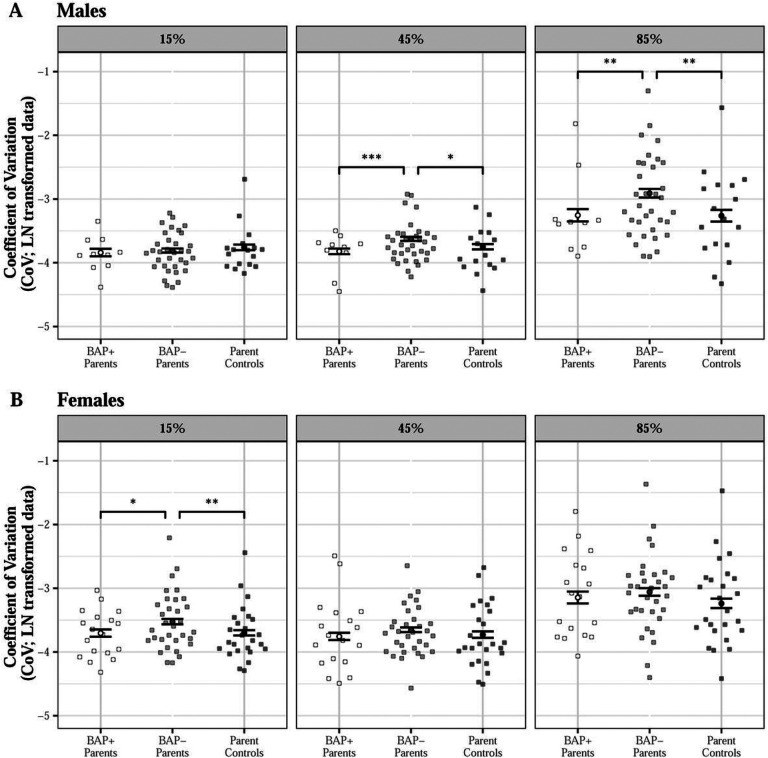
Force variability for proband parents and parent controls across force levels as a function of broader autism phenotype (BAP) classification and sex. BAP- fathers showed higher sustained force variability relative to BAP+ fathers (45% MVC: d=0.452; 85% MVC: d=0.380) and parent control males (45% MVC: d=0.264; 85% MVC: d=0.356) at 45% and 85% MVC (A) and BAP- mothers showed higher sustained force variability relative to parent control females (d=0.297) and BAP+ mothers (d=0.280) at 15% MVC [B; group (BAP- vs Control) × MVC × sex: β=−0.028, SE=0.010, p=0.004]. *p<0.05; **p < 0.01; ***p < 0.001.

Proband parents showed greater force variability than parent controls at 85% MVC with their dominant hand (d = 0.378) and at 45% MVC with their non-dominant hand (d = 0.285; group × MVC × hand: β = −0.018, SE = 0.009, p = 0.044, Figure S6). Proband fathers showed greater variability relative to male parent controls at 85% MVC only (d = 0.275; group × MVC × sex: β=−0.019, SE = 0.009, p = 0.043). These findings were driven by BAP- fathers showing greater force variability than male parent control (45% MVC: d = 0.264; 85% MVC: d = 0.356) and BAP + fathers at higher force levels [45% MVC: d = 0.452; 85% MVC: d = 0.380; group (BAP- vs control) × MVC × sex: β=−0.028, SE = 0.010, p = 0.004; group (BAP + vs control) × MVC × sex: β = 0.0004, SE = 0.012, p = 0.976; BAP + vs BAP- males at 45% MVC: t = 3.600, p < 0.001; 85% MVC: t = 2.929, p = 0.004); Fig. 8A]. BAP- mothers showed greater force variability than female parent controls (d = 0.297) and BAP + mothers at 15% MVC [d = 0.280; (BAP + vs BAP- mothers at 15% MVC: t = 2.538, p = 0.012; Fig. 8B).

#### Smooth Pursuit Gain

**Figure 9:**
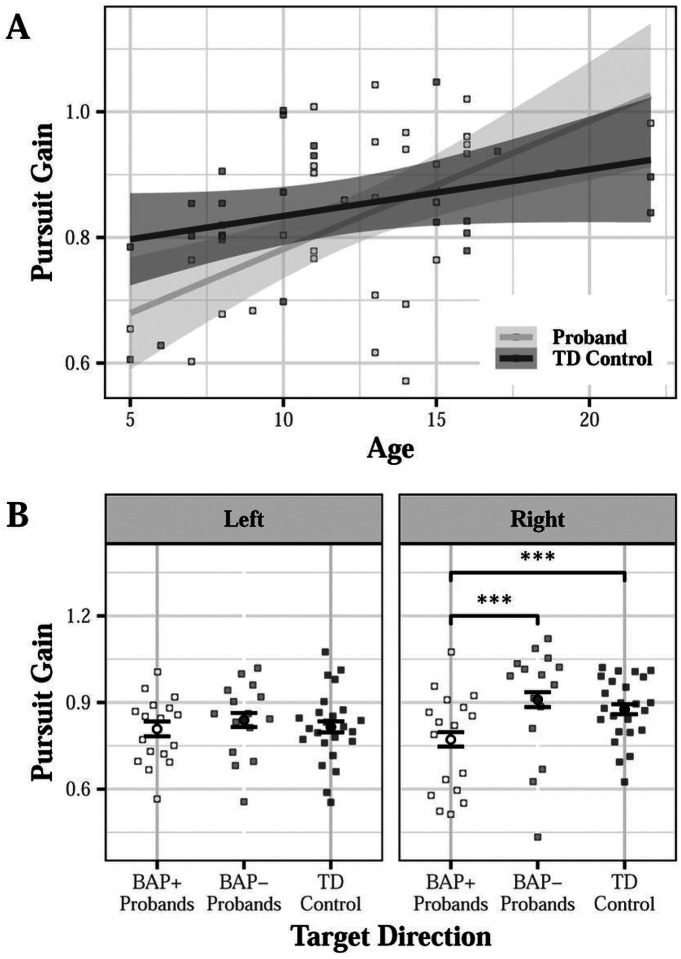
Pursuit gain for probands and TD controls as a function of age (A) and across target directions for probands as a function of broader autism phenotype (BAP) classification (B). BAP+ probands showed reduced pursuit gain compared to BAP- probands (d=0.410) and TD controls (d=0.263) for rightward trials [group (BAP+ vs control) × direction: β=−0.797, SE=0.278, p=0.004]. ***p<0.001. A marginally significant group × age interaction suggests that younger probands showed reduced pursuit gain compared to younger TD controls, but older probands showed similar or elevated pursuit gain compared to older controls (A; χ^2^(1)=3.703, p=0.054). B;

The group × age interaction approached significance for pursuit gain reflecting a reduction in pursuit gain in probands relative to controls at younger ages (d = 0.018; β = 0.054, SE = 0.028, p = 0.065; Fig. 9A). BAP + probands showed reduced pursuit gain compared to BAP- probands (d = 0.410) and TD controls (d = 0.263) for rightward trials only [group (BAP- vs control) × direction: β = 0.056, SE = 0.312, p = 0.858; group (BAP + vs control) × direction: β=−0.797, SE = 0.278, p = 0.004; BAP + vs BAP for rightward trials: t=−3.845, p < 0.001; Fig. 9B]. No differences in pursuit gain were seen between proband parents and parent controls or between BAP + or BAP- parents and parent controls.

#### Familiality of Sensorimotor De cits in Family Trios and Duos

Saccade velocity was highly intercorrelated among probands and their parents (h^2^ = 0.848, p < 0.001). Separate analyses for BAP + and BAP- families indicated that saccade velocity was familial across both groups (BAP+: h^2^ = 0.625, p = 0.036; BAP-: h^2^ = 0.953, p < 0.001); saccade duration was familial only for BAP- families (h^2^ = 0.557, p = 0.029). Precision gripping and pursuit outcomes were not familial.

#### Relationship Between Manual Motor and Oculomotor Behavior

For TD controls, increased rapid force overshooting at 15% MVC was related to increased saccade velocity (r = 0.579, p = 0.002; Table S3). For autistic individuals, increased pursuit gain was related to reduced grip force variability (r=−0.541, p = 0.002) and increased rapid grip force accuracy at 85% MVC (r = 0.522, p = 0.003). No other relationships between precision gripping and oculomotor outcomes were significant.

#### Clinical and Demographic Correlations

BAP + probands showed more severe parent-reported hyperactivity/impulsivity (t=−2.76, p = 0.01; Table 2) and trended toward showing more severe RBS-R rated ritualistic behaviors (t=−1.968, p = 0.056) and insistence on sameness (t=−1.946, p = 0.059) compared to BAP- probands. Neither precision gripping nor oculomotor impairments were related to ASD severity in probands or BAP features in parents (|r|’s ≤ 0.475; see Tables S4-S7).

## Discussion

We replicated our previous findings of feedforward and feedback sensorimotor impairments in autistic individuals [17, 20–22] and found that probands and their unaffected biological parents show similar patterns of sensorimotor control difficulties relative to neurotypical individuals which extends prior family studies [13, 19] in four key ways. First, we show that sensorimotor behaviors are impacted in unaffected parents across oculomotor and skeletomotor systems for rapid and sustained sensorimotor behaviors. Second, we found that sensorimotor difficulties were more severe in BAP- parents suggesting that sensorimotor impairments and BAP traits are familial characteristics that each confer unique risk for ASD. Third, rapid sensorimotor behaviors were more severely disrupted in BAP- probands across effectors while sustained behaviors were selectively impacted in BAP + probands. Finally, atypical saccade dynamics were highly familial, particularly between BAP- parents and their children, indicating that they may represent inherited liabilities in a subgroup of affected individuals without parental BAP traits. These results provide new evidence for multiple sensorimotor endophenotypes in ASD and highlight the complex interactions with liabilities reflected by parental autistic traits.

### Rapid and Sustained Sensorimotor Behavior in Probands and Parents

Probands and their biological parents showed similar impairments in rapid sensorimotor behaviors relative to controls implicating difficulties utilizing internal action representations to execute precise motor behaviors before sensory feedback information is available. This feedforward motor control dysfunction is more severely impacted in ASD families without parental BAP features. These novel findings suggest that rapid sensorimotor difficulties may represent inherited pathogenic risk processes that are independent from the risk accounted for by the presence of parental BAP traits [40, 41].

The familiality of these traits is reflected in our findings that saccade velocity and duration were highly inter-correlated among probands and their biological parents. Importantly, saccade dynamics were more strongly familial among BAP- parents and their children than in BAP + parents and their children, suggesting that they may be useful for separating the influence of different risk mechanisms for ASD. These results are consistent with research suggesting that inherited liability for ASD can be separated into independent components, including motor behaviors, attention, and social difficulties [18, 42, 43], and implicate rapid sensorimotor impairments as a new, distinct endophenotype useful for indexing familial liability with distinct ASD risk genes.

Our sustained sensorimotor outcomes indicated that utilizing sensory feedback for optimizing motor function is selectively impaired in BAP + probands and BAP- parents, suggesting overlapping or interacting risk with parental autistic traits. Further, BAP + probands showed more severe hyperactive/impulsive symptoms and repetitive behaviors compared to BAP- probands implicating a common inherited mechanism underlying clinical symptoms and impairments during sustained motor tasks which require higher attentional control compared to rapid motor tasks. Alternatively, as the majority of BAP + probands in our sample (77%) had one BAP + parent and one BAP- parent, sustained motor impairments in BAP + probands may reflect a combination of additive risk for ASD including familial autistic traits and sensorimotor feedback impairment, inherited separately from each parent. Studies assessing additive risk for ASD in offspring as a function of parental traits are needed to determine how sustained sensorimotor and autistic trait endophenotypes may interact to confer separable or joint risk.

### Limitations

While this study provides new insights into multiple familial endophenotypes associated with inherited risk for ASD, several limitations should be noted. First, many of our conclusions are drawn from relatively small samples of autistic individuals and their families. We emphasize the need for replication of these findings with larger samples of ASD families, including more autistic females, to address critical questions regarding independent or interactive familial risk factors conferred by separate sensorimotor and other endophenotypes. This also includes larger family trio samples with a greater number of probands with BAP + parents. Further, while a replication sample was not explicitly included in this study, these results extend previous findings from our group using separate samples examining manual motor [22, 44] and oculomotor control [20, 21]. Additionally, while family studies can offer evidence supporting inherited liabilities, genetic studies are needed to link endophenotype findings directly to genes. Finally, further examination of sensorimotor impairments as endophenotypes in early childhood may support earlier identification of ASD risk and targeted early intervention for motor impairments.

## Conclusions

Our findings identify rapid and sustained sensorimotor behaviors as discrete processes that are impaired in probands and their unaffected parents, affect multiple effector systems, and differentially relate to parental BAP traits. Findings that rapid motor difficulties are selectively impacted in BAP- probands and parents and are familial suggest that they may confer unique risk of ASD, independent of autistic trait inheritance, while sustained sensorimotor behaviors may jointly confer risk with BAP traits. Collectively, these results add to the growing literature suggesting quantitative trait dimensions outside of core autism traits may represent important endophenotypes useful for understanding distinct heritable risk pathways for ASD.

## Figures and Tables

**Table 1:** Demographic and clinical characteristics of participants.

	Probands (N = 58)	TD Controls (N = 42)	Proband Parents (N = 109)	Parent Controls (N = 47)
Age (years)	11 (4)	13 (6)	41 (6)	40 (6)
PIQ	103 (18)	106 (15)	111 (11)	109 (12)
VIQ	95 (19)***	110 (14)***	108 (12)	105 (12)
*Biological Sex* ^a^				
Female	9%**	31%**	53%	62%
Male	91%**	69%**	47%	36%

Note. Data are reported as mean and standard deviation in parentheses.

a Biological sex was compared across groups using a chi-square test.

Biological sex is not reported for one parent control. PIQ = performance IQ; VIQ = verbal IQ.

*** p < 0.001,

** p < 0.01.

**Table 2:** Demographic and clinical characteristics of probands and parents based on BAP status

	BAP + Probands (N = 22)	BAP− Probands (N = 23)	BAP+ Parents (N = 30)	BAP− Parents (N = 74)
% Male^a^	95	91	43	49
Age (years)	11 (4)	11 (3)	41 (5)	42 (6)
PIQ	104 (20)	100 (17)	113 (11)	110 (11)
VIQ	93 (19)	96 (20)	111 (13)	107 (11)
ADOS-2 CSS	8 (2)	8 (2)		
ADI-R Social	23 (5)	21 (6)		
ADI-R Communication	17 (5)	17 (4)		
ADI-R RRB	6 (3)	5 (2)		
RBS-R – Total	32 (16)	23 (17)		
RBS-R – Compulsive	4 (5)	3 (4)		
RBS-R – Restricted	4 (2)	3 (2)		
RBS-R – Ritualistic	7 (4)^~^	5 (4)		
RBS-R – Sameness	9 (5)^~^	6 (5)		
RBS-R – Self-Injurious	2 (3)	2 (2)		
RBS-R – Stereotyped	5 (3)	4 (4)		
Conners – Hyperactivity	76 (11)**	64 (14)		
BAP-Q Self Total			3.5 (0.6)***	2.4 (0.5)
BAP-Q Self Aloof			3.7 (0.8)***	2.5 (0.7)
BAP-Q Self Pragmatic			3.0 (0.8) ***	2.0 (0.5)
BAP-Q Self Rigid			3.7 (0.8) ***	2.6 (0.6)

Note. Data are reported as mean and standard deviation in parentheses.

a % Male was compared across groups using a chi-square test.

PIQ = performance IQ; VIQ = verbal IQ; ADOS-2 = Autism Diagnostic Observation Schedule, Second Edition; RBS-R = Repetitive Behavior Scale-Revised; BAP-Q = Broad Autism Phenotype-Questionnaire.

*** p < 0.001,

** p < 0.01,

~ p < 0.06.

## Data Availability

The datasets used and/or analyzed during the current study are available from the corresponding author upon reasonable request.

## Abbreviations

ADI-R: Autism Diagnostic Inventory – Revised
ADOS-2: Autism Diagnostic Observation Schedule, Second Edition
ASD: Autism Spectrum Disorder
BAP: Broader autism phenotypic traits
BAP−: Parent of autistic individual who does not demonstrate BAP features
BAP+: Parent of autistic individual who demonstrates BAP features
BAP-Q: Broad Autism Phenotype-Questionnaire
CoV: Coefficient of variation
CSS: Calibrated severity scale
DSM-5: Diagnostic and Statistical Manual of Mental Disorders, Fifth Edition
IQ: Intelligence quotient
MLM: Multilevel models
MVC: Maximum voluntary contraction
PANESS: Physical and Neurological Examination for Soft Signs
RBS-R: Repetitive Behavior Scale-Revised
SCQ: Social Communication Questionnaire
SOLAR: Sequential Oligogenic Linkage Analysis Routines
TD: Typically developing
VGS: Visually guided saccade
